# MIS‐C Overlap, Not Prior COVID‐19 Itself, Drives Treatment Escalation in Kawasaki Disease: A Nationwide Cohort Study

**DOI:** 10.1111/ped.70476

**Published:** 2026-07-13

**Authors:** Min Ji Kim, Jong Seung Kim, Jee Young Hong, Jihye You

**Affiliations:** ^1^ Department of Medical Informatics Jeonbuk National University Medical School Jeonju Korea; ^2^ Department of Otorhinolaryngology‐Head and Neck Surgery Jeonbuk National University Hospital Jeonju Korea; ^3^ Department of Preventive Medicine, College of Medicine Jeonbuk National University Jeonju Korea; ^4^ Research Institute of Clinical Medicine of Jeonbuk National University Jeonju Korea; ^5^ Department of Pediatrics Jeonbuk National University Children's Hospital Jeonju Korea

**Keywords:** COVID‐19, intravenous immunoglobulins, Kawasaki disease, multisystem inflammatory syndrome in children, SARS‐CoV‐2

## Abstract

**Background:**

Whether prior coronavirus disease 2019 (COVID‐19) independently influences treatment response in children with Kawasaki disease (KD) is unclear, as prior reports may have been confounded by overlap with multisystem inflammatory syndrome in children (MIS‐C). We investigated this in a nationwide cohort, accounting for these factors.

**Methods:**

We conducted a retrospective cohort study using the Korean National Health Insurance Service database. Children diagnosed with KD on or after January 1, 2020 who received IVIG within 7 days were included. Prior COVID‐19 was defined as documented infection within 3 months before KD diagnosis. The primary outcome was second‐line treatment within 7 days after initial IVIG (composite of second IVIG or additional steroid). 1:1 propensity score matching was performed using age, sex, MIS‐C status, and steroid use with initial IVIG.

**Results:**

Among 6572 eligible children, 512 had prior COVID‐19; 431 patients were matched per group. Second‐line treatment occurred in 32/431 (7.4%) versus 23/431 (5.3%) (*p* = 0.265). Prior COVID‐19 was not independently associated with second‐line treatment (adjusted HR 1.42; 95% CI 0.83–2.43). In subgroup analysis, elevated risk was confined to children with concurrent MIS‐C (HR 3.01; 95% CI 1.34–6.74); prior COVID‐19 without MIS‐C showed no increase (HR 1.18; 95% CI 0.66–2.11). Sensitivity analyses excluding MIS‐C and restricted to 2022 onward were consistent.

**Conclusions:**

Prior COVID‐19 alone was not independently associated with second‐line treatment once MIS‐C overlap and concurrent steroid use were accounted for. The elevated treatment escalation rate appeared driven primarily by MIS‐C, highlighting the importance of distinguishing MIS‐C from classic KD.

## 1 Introduction

Kawasaki disease (KD) is an acute vasculitis that primarily affects children younger than 5 years and remains the leading cause of acquired heart disease in this age group [1, 2, 3]. Although its precise etiology remains unclear, KD is widely considered to reflect an abnormal immune response to infectious triggers in genetically susceptible children [4, 5]. Early treatment with intravenous immunoglobulin (IVIG) substantially reduces the risk of coronary artery complications; however, a subset of patients requires additional treatment after the initial IVIG infusion, reflecting a more severe inflammatory course [6].

Since the coronavirus disease 2019 (COVID‐19) pandemic, the interpretation of pediatric inflammatory diseases has become more complex. Severe acute respiratory syndrome coronavirus 2 (SARS‐CoV‐2) infection has been linked to multisystem inflammatory syndrome in children (MIS‐C), which shares several clinical and immunologic features with KD, including persistent fever, mucocutaneous manifestations, and cardiovascular involvement [7, 8, 9, 10, 11]. This overlap has raised concern regarding whether inflammatory presentations diagnosed as KD after SARS‐CoV‐2 infection may differ in severity or treatment response from conventional KD.

South Korea provides a valuable setting for evaluating this question because KD incidence is high and national COVID‐19 surveillance has been comprehensive, enabling reliable identification of prior SARS‐CoV‐2 infection in a nationwide pediatric cohort [12, 13, 14]. Several studies have suggested that KD after SARS‐CoV‐2 infection may be associated with stronger inflammatory features, although available data remain limited and largely derived from single‐center or relatively small cohorts [11, 12, 15, 16]. In particular, population‐based evidence regarding treatment outcomes in children diagnosed with KD after prior COVID‐19 is still lacking, and the relative contributions of prior COVID‐19 itself versus concurrent MIS‐C remain unresolved.

Therefore, we used a nationwide cohort database to investigate whether prior COVID‐19 was independently associated with second‐line treatment after initial IVIG in children diagnosed with KD, after accounting for MIS‐C overlap and concurrent steroid use during initial treatment.

## 2 Methods

### 2.1 Data Source and Study Population

This nationwide retrospective cohort study used data from the Korean National Health Insurance Service (NHIS) database (NHIS‐2024‐1‐229). The NHIS is a population‐based claims database that includes diagnostic codes, procedures, prescriptions, and demographic information for the entire Korean population [17]. We identified children diagnosed with KD using the International Classification of Diseases, 10th Revision (ICD‐10) code M30.3 on or after January 1, 2020, and evaluated whether prior COVID‐19 was associated with second‐line treatment after initial IVIG.

We screened all eligible patients in the NHIS‐2024‐1‐229 database. To define an actively treated KD cohort, we included only patients who received at least 1 IVIG treatment within 7 days after the first KD diagnosis date. Nationwide COVID‐19 claims records were available in the database from October 8, 2020, to October 29, 2022. Patients without IVIG treatment within 7 days after KD diagnosis were excluded. We also excluded patients diagnosed with COVID‐19 within 1 week after KD diagnosis to avoid misclassification of exposure during the follow‐up period.

### 2.2 Exposure, Outcome, and Covariates

The exposure group consisted of children with documented COVID‐19 within 3 months before the KD diagnosis date. The control group consisted of children with KD who had no prior COVID‐19 diagnosis during that exposure window. Because nationwide COVID‐19 records were available from October 8, 2020, onward, exposure assessment was based on available claims records within the study period.

The primary outcome was second‐line treatment within 7 days after initial IVIG, defined as a composite of (a) second IVIG administration, identified as a repeat IVIG claim after the date of the initial IVIG, or (b) additional steroid treatment, defined as systemic steroid administration on a date later than the initial IVIG. Steroid use concurrent with initial IVIG (administered on the same day as the first IVIG claim) was defined as a baseline characteristic (‘steroid use with initial IVIG’) rather than as second‐line treatment. We acknowledge that this composite endpoint reflects clinical decision‐making and may not perfectly correspond to biologic IVIG resistance. To better characterize the components of the composite outcome, we additionally analyzed second IVIG and additional steroid as separate outcomes (Tables S1 and S2; Figures S1 and S2).

Baseline covariates included age, sex, economic status, residential area, neuromuscular disease, seizure disorder, MIS‐C status, and steroid use with initial IVIG. Neuromuscular disease was identified using ICD‐10 codes G50, G70, G71, G72, G73, and R56, and seizure disorder was identified using ICD‐10 codes G40 and G41. MIS‐C was defined using ICD‐10 code U10 within ±3 months of the KD diagnosis date. Because the diagnosis of MIS‐C may rely on serologic or clinical evidence of recent SARS‐CoV‐2 exposure rather than confirmed PCR positivity, a small number of MIS‐C cases without a documented COVID‐19 surveillance entry were observed in the no‐prior‐COVID‐19 group, likely reflecting incomplete capture of asymptomatic infection.

### 2.3 Statistical Analysis

To balance baseline characteristics between groups, we performed 1:1 propensity score matching between children with prior COVID‐19 and controls. Propensity scores were estimated using a logistic regression model that included age, sex, MIS‐C status, and steroid use with initial IVIG as matching variables. Greedy 1:1 nearest‐neighbor matching was applied. Standardized mean differences (SMDs) were used to assess covariate balance, with values ≤ 0.10 considered well‐balanced. After matching, 431 patients were included in each group.

Categorical variables were compared using the chi‐square test or Fisher exact test, as appropriate. Continuous variables were compared using the Student *t*‐test or Wilcoxon rank‐sum test, depending on distribution.

Time‐to‐event analyses for second‐line treatment during the 7‐day follow‐up period were performed using Cox proportional hazards regression. A time‐to‐event approach was selected over a binary logistic model because the timing of second‐line treatment varied meaningfully within the 7‐day window, and the Cox framework appropriately handles partial follow‐up when no event occurred by day 7. The multivariable model was pre‐specified and included COVID‐19 status, age (< 3 years vs. ≥ 3 years), sex, MIS‐C status, steroid use with initial IVIG, neuromuscular disease, economic status, and residential area. Seizure disorder, although pre‐specified as a covariate, was excluded from the multivariable model post hoc because of non‐convergence due to zero events in a stratum. Hazard ratios (HRs) with 95% confidence intervals (CIs) were calculated.

Sensitivity analyses were performed to address potential confounding from MIS‐C overlap and exposure misclassification. First, the main analysis was repeated after excluding all children with MIS‐C. Second, the analysis was restricted to children diagnosed with KD on or after January 1, 2022, when the Korean nationwide COVID‐19 surveillance system was relatively stable.

A two‐sided *p* value < 0.05 was considered statistically significant.

### 2.4 Ethics Statement

The study protocol was reviewed and approved by the Institutional Review Board of Jeonbuk National University Hospital (approval No. 2023‐02‐041). The requirement for informed consent was waived because this study involved a research review of national insurance data. This study was conducted in accordance with the principles outlined in the Declaration of Helsinki.

## 3 Results

### 3.1 Study Population and Baseline Characteristics

**FIGURE 1:**
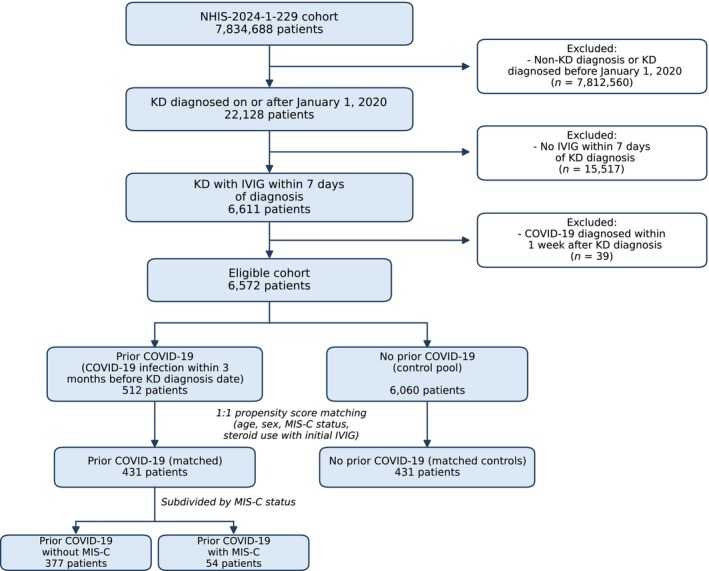
Flow diagram of study population selection. Among 7,834,688 individuals in the NHIS‐2024‐1‐229 database, 22,128 children were diagnosed with Kawasaki disease (KD) on or after January 1, 2020. After excluding patients who did not receive intravenous immunoglobulin (IVIG) within 7 days after KD diagnosis and those diagnosed with coronavirus disease 2019 (COVID‐19) within 1 week after KD diagnosis, 6572 patients remained eligible for analysis. Among them, 512 children had documented COVID‐19 within 3 months before KD diagnosis, whereas 6060 children had no prior COVID‐19. After 1:1 propensity score matching including age, sex, MIS‐C status, and steroid use with initial IVIG as matching variables, 431 patients were included in each group. Within the matched prior COVID‐19 group, 377 were classified as KD without MIS‐C and 54 were classified as KD with concurrent MIS‐C.

Among 7,834,688 individuals in the NHIS‐2024‐1‐229 database, 22,128 children were diagnosed with KD on or after January 1, 2020. After excluding patients who did not receive IVIG within 7 days after KD diagnosis and those diagnosed with COVID‐19 within 1 week after KD diagnosis, 6572 patients remained eligible for analysis. Among them, 512 children had documented COVID‐19 within 3 months before KD diagnosis, whereas 6060 had no prior COVID‐19. Baseline characteristics before propensity score matching are summarized in Table S3. After 1:1 propensity score matching including age, sex, MIS‐C status, and steroid use with initial IVIG as matching variables, 431 patients were included in each group. Within the matched cohort, 54 of 431 children (12.5%) in the prior COVID‐19 group had concurrent MIS‐C, while 52 of 431 children (12.1%) in the matched control group had a recorded MIS‐C diagnosis without a documented COVID‐19 surveillance entry (Figure 1). Baseline characteristics of the matched cohort showed excellent balance across all matching variables (all SMD ≤ 0.108; Table 1).

**TABLE 1:** Baseline characteristics of the matched cohort according to prior COVID‐19 status.

Variable	Prior COVID‐19 (*n* = 431)	No prior COVID‐19 (*n* = 431)	*p*	SMD
Male sex (%)	252 (58.5)	267 (61.9)	0.330	0.071
Age < 3 years (%)	240 (55.7)	245 (56.8)	0.784	0.023
Age (years), mean (SD)	3.95 (3.19)	3.84 (3.20)	0.624	0.033
Age (months), mean (SD)	47.39 (38.26)	46.11 (38.40)	0.624	0.033
High economic status (%)	148 (34.3)	163 (37.8)	0.321	0.073
Metropolitan residence (%)	199 (46.2)	176 (40.8)	0.131	0.108
MIS‐C (%)	54 (12.5)	52 (12.1)	0.917	0.014
Steroid use with initial IVIG (%)	188 (43.6)	185 (42.9)	0.891	0.014
Neuromuscular disease (%)	53 (12.3)	43 (10.0)	0.330	0.074
Seizure disorder (%)	4 (0.9)	4 (0.9)	1.000	< 0.001
Prior KD within 1 year (%)	3 (0.7)	5 (1.2)	0.722	0.048
Prior MIS‐C within 1 year (%)	2 (0.5)	1 (0.2)	1.000	0.039
Second IVIG (%)	16 (3.7)	14 (3.2)	0.853	0.025
Additional steroid (%)	20 (4.6)	12 (2.8)	0.207	0.098
Second‐line treatment, composite (%)	32 (7.4)	23 (5.3)	0.265	0.086

*Note:* Data are presented as *n* (%) or mean (standard deviation), as appropriate. Propensity score matching included age, sex, MIS‐C status, and steroid use with initial IVIG as matching variables. After matching, all four matching variables showed standardized mean differences ≤ 0.10, indicating successful balance. Variables in red indicate revisions made in response to reviewer comments.

Abbreviations: IVIG, intravenous immunoglobulin; KD, Kawasaki disease; MIS‐C, multisystem inflammatory syndrome in children; SMD, standardized mean difference.

### 3.2 Association of Prior COVID‐19 With Second‐Line Treatment

**FIGURE 2:**
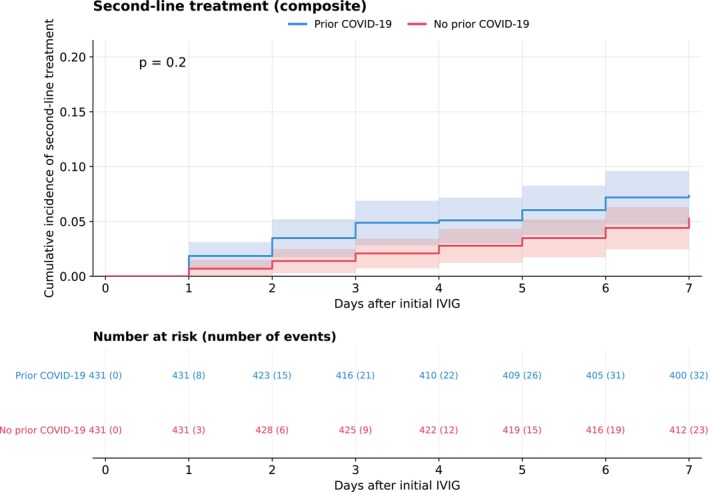
Cumulative incidence of second‐line treatment within 7 days according to prior COVID‐19 status in the matched cohort. After matching on age, sex, MIS‐C status, and steroid use with initial IVIG, the cumulative incidence of second‐line treatment did not differ significantly between children with prior COVID‐19 and matched controls (log‐rank *p* = 0.20). Shaded areas indicate 95% confidence intervals. Numbers at risk and cumulative numbers of events are shown below the plot.

Second‐line treatment within 7 days occurred in 32/431 (7.4%) children with prior COVID‐19 versus 23/431 (5.3%) matched controls (*p* = 0.265). Among the second‐line treatment events, second IVIG administration occurred in 16 (3.7%) versus 14 (3.2%), and additional steroid treatment in 20 (4.6%) versus 12 (2.8%), in the prior COVID‐19 and matched control groups, respectively. The cumulative incidence of second‐line treatment did not differ significantly between groups (log‐rank *p* = 0.20; Figure 2). In Cox regression analysis, prior COVID‐19 was not significantly associated with second‐line treatment in either univariable analysis (HR 1.42; 95% CI 0.83–2.43) or multivariable analysis (HR 1.42; 95% CI 0.83–2.43; *p* = 0.205) (Table 2; Table S4). When the composite outcome was decomposed, neither second IVIG alone (aHR 1.19; 95% CI 0.58–2.44; *p* = 0.634) nor additional steroid alone (aHR 1.66; 95% CI 0.81–3.42; *p* = 0.170) showed a statistically significant association with prior COVID‐19 (Tables S1 and S2; Figures S1 and S2).

**TABLE 2:** Association of prior COVID‐19 with second‐line treatment in the matched cohort.

Variable	Events/Total (%)	Univariable HR (95% CI)	Multivariable HR (95% CI)	*p*
No prior COVID‐19	23/431 (5.3)	Reference	Reference	—
Prior COVID‐19	32/431 (7.4)	1.42 (0.83–2.43)	1.42 (0.83–2.43)	0.205

*Note:* Multivariable Cox regression was adjusted for age (< 3 years vs. ≥ 3 years), sex, MIS‐C status, steroid use with initial IVIG, neuromuscular disease, economic status, and residential area. Seizure disorder was excluded from the multivariable model due to non‐convergence (zero events in a stratum). A time‐to‐event approach using Cox proportional hazards regression was selected because the timing of second‐line treatment varied meaningfully within the 7‐day window, and the Cox framework appropriately handles partial follow‐up when no event occurred by day 7.

Abbreviations: CI, confidence interval; HR, hazard ratio.

### 3.3 Subgroup Analysis According to MIS‐C Status

**FIGURE 3:**
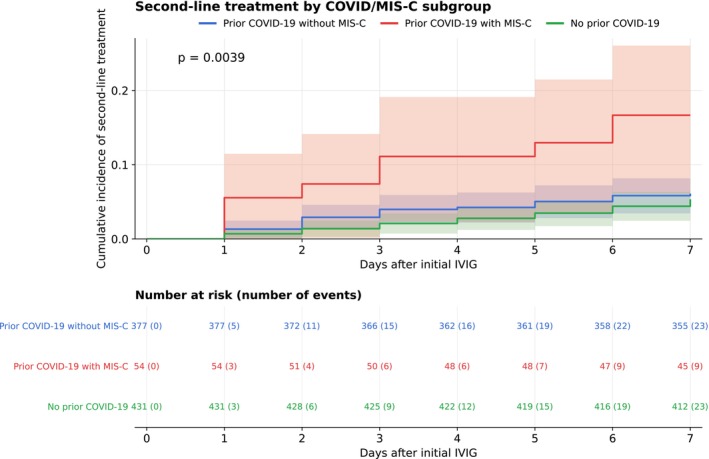
Cumulative incidence of second‐line treatment within 7 days according to prior COVID‐19 and MIS‐C status (three‐way subgroup analysis). Children with prior COVID‐19 and concurrent MIS‐C (*n* = 54) showed a substantially higher cumulative incidence of second‐line treatment than the other two groups (log‐rank *p* = 0.004). The cumulative incidence in children with prior COVID‐19 without MIS‐C (*n* = 377) closely approximated that of matched controls without prior COVID‐19 (*n* = 431). Shaded areas indicate 95% confidence intervals. Numbers at risk and cumulative numbers of events are shown below the plot.

We performed a three‐way subgroup analysis comparing matched controls (*n* = 431), prior COVID‐19 without MIS‐C (*n* = 377), and prior COVID‐19 with MIS‐C (*n* = 54); clinical characteristics of these three groups are summarized in Table S5. The cumulative incidence of second‐line treatment was highest among children with prior COVID‐19 and MIS‐C, while the cumulative incidence in children with prior COVID‐19 without MIS‐C closely approximated that of matched controls without prior COVID‐19 (log‐rank *p* = 0.004; Figure 3). In Cox regression analysis using no prior COVID‐19 as the reference group, prior COVID‐19 without MIS‐C was not significantly associated with second‐line treatment (HR 1.18; 95% CI 0.66–2.11; *p* = 0.572). In contrast, prior COVID‐19 with MIS‐C was strongly associated with an increased risk of second‐line treatment (HR 3.01; 95% CI 1.34–6.74; *p* = 0.007) (Table S6).

### 3.4 Sensitivity Analyses

Sensitivity analyses supported the main findings. After excluding all children with MIS‐C, prior COVID‐19 was not significantly associated with second‐line treatment (aHR 1.19; 95% CI 0.65–2.16; *p* = 0.579) (Table S7). Restricting to children diagnosed with KD on or after January 1, 2022, when COVID‐19 surveillance was most stable, also did not reveal a significant association (aHR 1.39; 95% CI 0.64–3.03; *p* = 0.406) (Table S8). Temporal trends in monthly COVID‐19 and Kawasaki disease case counts during the study period are shown in Figure S3.

## 4 Discussion

In this nationwide cohort study of children diagnosed with KD in South Korea, prior COVID‐19 alone was not independently associated with second‐line treatment after initial IVIG once MIS‐C overlap and concurrent steroid use were accounted for in the matching process. Although our preliminary analysis matched only on age and sex had suggested an approximately 2‐fold higher hazard of treatment escalation in the prior COVID‐19 group, this association was substantively attenuated after the propensity score model was extended to include MIS‐C status and steroid use with initial IVIG (aHR 1.42; 95% CI 0.83–2.43; *p* = 0.205). In a three‐way subgroup analysis, the elevated risk was confined to children with concurrent MIS‐C (aHR 3.01; 95% CI 1.34–6.74; *p* = 0.007), whereas children with prior COVID‐19 without MIS‐C showed no significant increase (aHR 1.18; 95% CI 0.66–2.11; *p* = 0.572). This pattern was consistent in sensitivity analyses excluding MIS‐C cases (aHR 1.19; 95% CI 0.65–2.16; *p* = 0.579) and restricting to the stable COVID‐19 capture period (2022 onward; aHR 1.39; 95% CI 0.64–3.03; *p* = 0.406), supporting the robustness of this interpretation. Our findings therefore indicate that the previously observed association between prior COVID‐19 and treatment escalation was largely driven by MIS‐C overlap rather than by prior SARS‐CoV‐2 infection itself increasing refractoriness in classic KD.

This study has clinical relevance because it used nationwide claims data linked to a comprehensive COVID‐19 surveillance system in South Korea [18, 19]. This setting allowed population‐level identification of both prior COVID‐19 and IVIG‐treated KD [19]. In addition, because IVIG treatment for KD is closely tied to insurance claims records, misclassification among actively treated patients may have been reduced [17]. These features may improve the interpretability of the observed association between prior COVID‐19 and second‐line treatment.

An important observation in this study was that the cumulative incidence of second‐line treatment was strikingly elevated in children with prior COVID‐19 and MIS‐C, while children with prior COVID‐19 without MIS‐C had a treatment escalation rate similar to matched controls without prior COVID‐19. This pattern is consistent with the possibility that diagnostic and inflammatory overlap between KD and MIS‐C is the principal driver of the observed association, rather than prior SARS‐CoV‐2 infection per se directly worsening classic KD [20, 21]. In real‐world practice, children diagnosed with KD after recent COVID‐19 represent a clinically heterogeneous group that includes post‐COVID inflammatory phenotypes such as MIS‐C with different treatment responses [21, 22]. Our findings emphasize that careful distinction between classic KD and MIS‐C is essential when interpreting treatment outcomes in the post‐COVID era.

Our findings should also be interpreted in the context of changes in clinical practice during and after the COVID‐19 pandemic. Previous studies have reported changes in KD incidence during the pandemic, including reduced case numbers in association with social distancing and other behavioral changes [15, 16, 23]. In our dataset, monthly KD case counts showed relatively modest temporal variation despite marked fluctuations in COVID‐19 incidence, suggesting that the association observed in this study may not be explained solely by temporal changes in overall disease occurrence. In addition, concurrent steroid use during initial treatment was frequent, particularly among children with prior COVID‐19 and MIS‐C, which may reflect evolving practice patterns and increased clinician concern regarding MIS‐C or other severe inflammatory presentations [24, 25]. Once concurrent steroid use was incorporated into the propensity score model, the apparent association between prior COVID‐19 and treatment escalation no longer reached statistical significance, suggesting that earlier reports of an association may have been confounded by clinician behavior toward suspected MIS‐C cases.

The biological basis of this association cannot be determined from administrative data alone. Although prolonged immune activation after SARS‐CoV‐2 infection has been proposed in previous studies [22, 26], our data do not establish a direct mechanistic role for SARS‐CoV‐2 antibodies or demonstrate that prior COVID‐19 independently worsens classic KD. Rather, the present findings are more appropriately understood as reflecting a population‐level association between MIS‐C overlap and treatment escalation among children coded as having KD after recent COVID‐19 [20, 21].

Several limitations should be considered. First, the retrospective design and use of administrative claims data may have introduced misclassification in exposure, phenotype, and outcome definitions. Second, second‐line treatment is a pragmatic treatment‐based endpoint and may not fully correspond to biologically defined IVIG resistance. Third, clinical details such as laboratory findings, echocardiographic results, and symptom profiles were unavailable, limiting the ability to distinguish classic KD from MIS‐C precisely. Fourth, exposure misclassification is an inherent concern when using nationwide COVID‐19 surveillance records. The Korean COVID‐19 surveillance system was actively maintained primarily from 2020 through 2022; routine PCR‐based mass screening was scaled back in 2023, and asymptomatic or unreported infections may not have been captured throughout the study period. As a result, the control group may have included children with undocumented prior SARS‐CoV‐2 infection (non‐differential misclassification), which would tend to bias the estimated association toward the null. To mitigate this concern, we performed a sensitivity analysis restricted to the period after January 1, 2022, when surveillance capture was relatively stable, and the results were consistent with the main analysis (aHR 1.39; 95% CI 0.64–3.03; *p* = 0.406). Finally, treatment decisions, including adjunctive steroid use, may have been influenced by clinician preference and evolving post‐pandemic practice patterns.

In conclusion, prior COVID‐19 alone was not independently associated with second‐line treatment after initial IVIG when MIS‐C overlap and concurrent steroid use were accounted for in the matching process. The elevated treatment escalation rate previously observed in children with prior COVID‐19 appeared to be driven primarily by concurrent MIS‐C, rather than by prior SARS‐CoV‐2 infection itself. These findings emphasize the importance of distinguishing MIS‐C from classic KD in clinical evaluation and underscore the need for further studies integrating detailed clinical and immunologic data to clarify the contribution of post‐COVID inflammatory phenotypes to KD treatment outcomes.

Supplementary analyses supporting these findings, including the separate‐outcome and subgroup regression models, the baseline comparison before propensity score matching, and the sensitivity analyses, together with the corresponding cumulative incidence curves and temporal case‐count trends, are provided as Tables S1–S8 and Figures S1–S3.

## Author Contributions

J.Y. and J.S.K. conceived and designed the study; M.J.K. curated the data and performed the formal analysis; J.Y. and M.J.K. conducted the investigation; J.Y., J.S.K., and J.Y.H. developed the methodology; J.Y. administered the project and provided resources; J.S.K. supervised and validated the study; M.J.K. visualized the data; J.Y. and M.J.K. drafted the manuscript; J.Y., M.J.K., J.S.K., and J.Y.H. reviewed and edited the manuscript. All authors read and approved the final manuscript.

## Funding

This work was supported by the Fund of the Biomedical Research Institute, Jeonbuk National University Hospital (2026).

## Disclosure

The authors have nothing to report.

## Conflicts of Interest

The authors declare no conflicts of interest.

## Supporting information


**Table S1:** Cox regression analysis for second IVIG administration alone in the matched cohort.
**Table S2:** Cox regression analysis for additional steroid treatment alone in the matched cohort.
**Table S3:** Baseline characteristics before propensity score matching according to prior COVID‐19 status.
**Table S4:** Univariable and multivariable Cox regression analyses of factors associated with second‐line treatment in the matched cohort.
**Table S5:** Clinical characteristics of the matched cohort according to COVID‐19 and MIS‐C status (three‐way comparison).
**Table S6:** Univariable and multivariable Cox regression analyses according to prior COVID‐19 and MIS‐C status in the matched cohort.
**Table S7:** Sensitivity analysis excluding all MIS‐C cases.
**Table S8:** Sensitivity analysis restricted to the stable COVID‐19 capture period (KD diagnosed in 2022 or later).
**Figure S1:** Cumulative incidence of second IVIG administration within 7 days according to prior COVID‐19 status in the matched cohort.
**Figure S2:** Cumulative incidence of additional steroid treatment within 7 days according to prior COVID‐19 status in the matched cohort.
**Figure S3:** Temporal trends in monthly COVID‐19 and Kawasaki disease case counts during the study period.

## Data Availability

The data that support the findings of this study are available on request from the corresponding author. The data are not publicly available due to privacy or ethical restrictions.
