# Supplementary material for: MIS‐C Overlap, Not Prior COVID‐19 Itself, Drives Treatment Escalation in Kawasaki Disease: A Nationwide Cohort Study
Source: Pediatr Int. 2026 Jul 13;68(1):e70476. doi: 10.1111/ped.70476 (PMC13359311; doi:10.1111/ped.70476)
Supplement: Supplementary file 1 — Table S1: Cox regression analysis for second IVIG administration alone in the matched cohort. Table S2: Cox regression analysis for additional steroid treatment alone in the matched cohort. Table S3: Baseline characteristics before propensity score matching according to prior COVID‐19 status. Table S4: Univariable and multivariable Cox regression analyses of factors associated with second‐line treatment in the matched cohort. Table S5: Clinical characteristics of the matched cohort according to COVID‐19 and MIS‐C status (three‐way comparison). Table S6: Univariable and multivariable Cox regression analyses according to prior COVID‐19 and MIS‐C status in the matched cohort. Table S7: Sensitivity analysis excluding all MIS‐C cases. Table S8: Sensitivity analysis restricted to the stable COVID‐19 capture period (KD diagnosed in 2022 or later). Figure S1: Cumulative incidence of second IVIG administration within 7 days according to prior COVID‐19 status in the matched cohort. Figure S2: Cumulative incidence of additional steroid treatment within 7 days according to prior COVID‐19 status in the matched cohort. Figure S3: Temporal trends in monthly COVID‐19 and Kawasaki disease case counts during the study period. [file PED-68-e70476-s001.docx]

**Supplementary Table 1. Cox regression analysis for second IVIG administration alone in the matched cohort.**

| **Subgroup** | **Events / total (%)** | **Univariable HR (95% CI)** | **Multivariable HR (95% CI)** | **P value** |
| --- | --- | --- | --- | --- |
| No prior COVID-19 | 14/431 (3.2) | Reference | Reference | — |
| Prior COVID-19 | 16/431 (3.7) | 1.16 (0.57–2.38) | 1.19 (0.58–2.44) | 0.634 |

*Total N = 862, 30 events. HR, hazard ratio; CI, confidence interval. Multivariable model adjusted for age (<3 years vs ≥3 years), sex, MIS-C status, steroid use with initial IVIG, neuromuscular disease, economic status, and residential area.*

**Supplementary Table 2. Cox regression analysis for additional steroid treatment alone in the matched cohort.**

| **Subgroup** | **Events / total (%)** | **Univariable HR (95% CI)** | **Multivariable HR (95% CI)** | **P value** |
| --- | --- | --- | --- | --- |
| No prior COVID-19 | 12/431 (2.8) | Reference | Reference | — |
| Prior COVID-19 | 20/431 (4.6) | 1.71 (0.83–3.49) | 1.66 (0.81–3.42) | 0.170 |

*Total N = 862, 32 events. HR, hazard ratio; CI, confidence interval. Additional steroid treatment refers to systemic steroid administration on a date later than the initial IVIG (excluding same-day steroid use, which was treated as a baseline covariate).*

*Multivariable model adjusted for age (<3 years vs ≥3 years), sex, MIS-C status, steroid use with initial IVIG, neuromuscular disease, economic status, and residential area.*

**Supplementary Table 3. Baseline characteristics before propensity score matching according to prior COVID-19 status.**

| **Variable** | **Prior COVID-19 (n = 512)** | **No prior COVID-19 (n = 6,060)** | **P value** | **SMD** |
| --- | --- | --- | --- | --- |
| Male sex (%) | 303 (59.2) | 3,474 (57.3) | 0.443 | 0.038 |
| Age <3 years (%) | 249 (48.6) | 4,615 (76.2) | <0.001 | 0.593 |
| Age (years), mean (SD) | 4.54 (3.49) | 2.43 (2.31) | <0.001 | 0.713 |
| Age (months), mean (SD) | 54.54 (41.88) | 29.22 (27.73) | <0.001 | 0.713 |
| High economic status (%) | 181 (35.4) | 2,092 (34.5) | 0.741 | 0.017 |
| Metropolitan residence (%) | 241 (47.1) | 2,544 (42.0) | 0.028 | 0.103 |
| MIS-C (%) | 134 (26.2) | 53 (0.9) | <0.001 | 0.796 |
| Steroid use with initial IVIG (%) | 258 (50.4) | 2,040 (33.7) | <0.001 | 0.344 |
| Neuromuscular disease (%) | 63 (12.3) | 537 (8.9) | 0.012 | 0.112 |
| Seizure disorder (%) | 6 (1.2) | 45 (0.7) | 0.423 | 0.044 |
| Prior KD within 1 year (%) | 4 (0.8) | 67 (1.1) | 0.646 | 0.034 |
| Prior MIS-C within 1 year (%) | 4 (0.8) | 1 (0.0) | <0.001 | 0.122 |
| Second IVIG (%) | 19 (3.7) | 167 (2.8) | 0.266 | 0.054 |
| Additional steroid (%) | 30 (5.9) | 167 (2.8) | <0.001 | 0.153 |
| Second-line treatment, composite (%) | 42 (8.2) | 294 (4.9) | 0.001 | 0.136 |

*Data are presented as n (%) or mean (standard deviation), as appropriate.*

*MIS-C cases in the no-prior-COVID-19 group represent children with an ICD-10 U10 diagnosis but no matched COVID-19 surveillance entry within the exposure window. This may occur because MIS-C diagnosis can rely on serologic or clinical evidence of recent SARS-CoV-2 exposure rather than confirmed PCR positivity, or because of incomplete capture of asymptomatic infection.*

*KD, Kawasaki disease; MIS-C, multisystem inflammatory syndrome in children; IVIG, intravenous immunoglobulin; SMD, standardized mean difference. Items in red indicate revisions made in response to reviewer comments.*

**Supplementary Table 4. Univariable and multivariable Cox regression analyses of factors associated with second-line treatment in the matched cohort.**

| **Variable** | **Univariable HR (95% CI)** | **Multivariable HR (95% CI)** | **P value (multivariable)** |
| --- | --- | --- | --- |
| Prior COVID-19 (vs No) | 1.42 (0.83–2.43) | 1.42 (0.83–2.43) | 0.205 |
| Age <3 years (vs ≥3) | 0.74 (0.44–1.26) | 0.84 (0.49–1.46) | 0.547 |
| Sex, Male (vs Female) | 1.00 (0.58–1.71) | 1.07 (0.62–1.85) | 0.797 |
| MIS-C (vs No) | 2.08 (1.10–3.94) | 1.82 (0.93–3.54) | 0.080 |
| Steroid use with initial IVIG (vs No) | 1.71 (1.00–2.91) | 1.54 (0.88–2.67) | 0.128 |
| Neuromuscular disease (vs No) | 1.16 (0.52–2.56) | 1.12 (0.50–2.48) | 0.789 |
| High economic status (vs Low) | 1.09 (0.63–1.88) | 1.01 (0.58–1.76) | 0.967 |
| Metropolitan residence (vs Rural) | 0.93 (0.55–1.59) | 0.85 (0.49–1.46) | 0.554 |

*Total N = 862 (431 prior COVID-19 + 431 matched controls); 55 events. HR, hazard ratio; CI, confidence interval; MIS-C, multisystem inflammatory syndrome in children.*

*Multivariable Cox regression included all variables shown in this table simultaneously (pre-specified model). Seizure disorder, although pre-specified as a covariate, was excluded from the multivariable model due to non-convergence (zero events in a stratum). The matched cohort included 431 children with prior COVID-19 and 431 matched controls.*

**Supplementary Table 5. Clinical characteristics of the matched cohort according to COVID-19 and MIS-C status (3-way comparison).**

| **Variable** | **No prior COVID-19 (n = 431)** | **COVID without MIS-C (n = 377)** | **COVID with MIS-C (n = 54)** | **P value** | **SMD** |
| --- | --- | --- | --- | --- | --- |
| Age <3 years (%) | 245 (56.8) | 222 (58.9) | 18 (33.3) | 0.002 | 0.353 |
| Age (months), mean (SD) | 46.11 (38.40) | 43.64 (35.29) | 73.56 (47.38) | <0.001 | 0.473 |
| Male sex (%) | 267 (61.9) | 231 (61.3) | 21 (38.9) | 0.004 | 0.316 |
| Steroid use with initial IVIG (%) | 185 (42.9) | 150 (39.8) | 38 (70.4) | <0.001 | 0.429 |
| Neuromuscular disease (%) | 43 (10.0) | 42 (11.1) | 11 (20.4) | 0.073 | 0.195 |
| Seizure disorder (%) | 4 (0.9) | 4 (1.1) | 0 (0.0) | 0.749 | 0.099 |
| Second IVIG (%) | 14 (3.2) | 15 (4.0) | 1 (1.9) | 0.679 | 0.085 |
| Additional steroid (%) | 12 (2.8) | 11 (2.9) | 9 (16.7) | <0.001 | 0.322 |
| Second-line treatment, composite (%) | 23 (5.3) | 23 (6.1) | 9 (16.7) | 0.006 | 0.246 |

*Data are presented as n (%) or mean (standard deviation), as appropriate.*

*Within the matched cohort (N=862), the prior COVID-19 group was further subdivided according to MIS-C status (377 children without MIS-C and 54 children with MIS-C). The no prior COVID-19 reference group included 431 matched controls. Note that the unmatched MIS-C numbers (134 in the COVID-19 group; 53 in the no prior COVID-19 group, see Supplementary Table 3) differ from the matched-cohort numbers shown here.*

*KD, Kawasaki disease; MIS-C, multisystem inflammatory syndrome in children; IVIG, intravenous immunoglobulin; SMD, standardized mean difference.*

**Supplementary Table 6. Univariable and multivariable Cox regression analyses according to prior COVID-19 and MIS-C status in the matched cohort.**

| **Subgroup** | **Univariable HR (95% CI)** | **Multivariable HR (95% CI)** | **P value** |
| --- | --- | --- | --- |
| No prior COVID-19 (n = 431) | Reference | Reference | — |
| Prior COVID-19 without MIS-C (n = 377) | 1.18 (0.66–2.11) | 1.18 (0.66–2.11) | 0.572 |
| Prior COVID-19 with MIS-C (n = 54) | 3.01 (1.34–6.74) | 3.01 (1.34–6.74) | 0.007 |

*Total N = 862, 55 events. HR, hazard ratio; CI, confidence interval; MIS-C, multisystem inflammatory syndrome in children.*

*Multivariable Cox regression was adjusted for age (<3 years vs ≥3 years), sex, steroid use with initial IVIG, neuromuscular disease, economic status, and residential area.*

**Supplementary Table 7. Sensitivity analysis excluding all MIS-C cases.**

| **Subgroup** | **Univariable HR (95% CI)** | **Multivariable HR (95% CI)** | **P value** |
| --- | --- | --- | --- |
| No prior COVID-19 (n = 379) | Reference | Reference | — |
| Prior COVID-19 without MIS-C (n = 377) | 1.17 (0.65–2.14) | 1.19 (0.65–2.16) | 0.579 |

*Total N = 756 (after excluding all 106 MIS-C cases); 43 events. HR, hazard ratio; CI, confidence interval. Multivariable model adjusted for age (<3 years vs ≥3 years), sex, steroid use with initial IVIG, neuromuscular disease, economic status, and residential area (MIS-C variable excluded since all participants are non-MIS-C).*

*This sensitivity analysis was performed in response to Reviewer Comment 1 (Major) to assess whether the association between prior COVID-19 and second-line treatment persists in classic KD without MIS-C overlap.*

**Supplementary Table 8. Sensitivity analysis restricted to the stable COVID-19 capture period (KD diagnosed in 2022 or later).**

| **Subgroup** | **Univariable HR (95% CI)** | **Multivariable HR (95% CI)** | **P value** |
| --- | --- | --- | --- |
| No prior COVID-19 (n = 133) | Reference | Reference | — |
| Prior COVID-19 (n = 420; 2022 onward) | 1.14 (0.54–2.39) | 1.39 (0.64–3.03) | 0.406 |

*Total N = 553 (KD diagnosed January 2022 onward); 41 events. HR, hazard ratio; CI, confidence interval. Multivariable model adjusted for age (<3 years vs ≥3 years), sex, MIS-C status, steroid use with initial IVIG, neuromuscular disease, economic status, and residential area.*

*This sensitivity analysis was performed in response to Reviewer Comment 4 (Major) to address potential exposure misclassification due to incomplete COVID-19 surveillance during the early pandemic period. The Korean nationwide COVID-19 surveillance was most stable during the Omicron-dominant period (from January 2022 onward), and analyses restricted to this period yielded results consistent with the main analysis.*

**Supplementary Figure Legends**

**Supplementary Figure 1. Cumulative incidence of second IVIG administration within 7 days according to prior COVID-19 status in the matched cohort.**


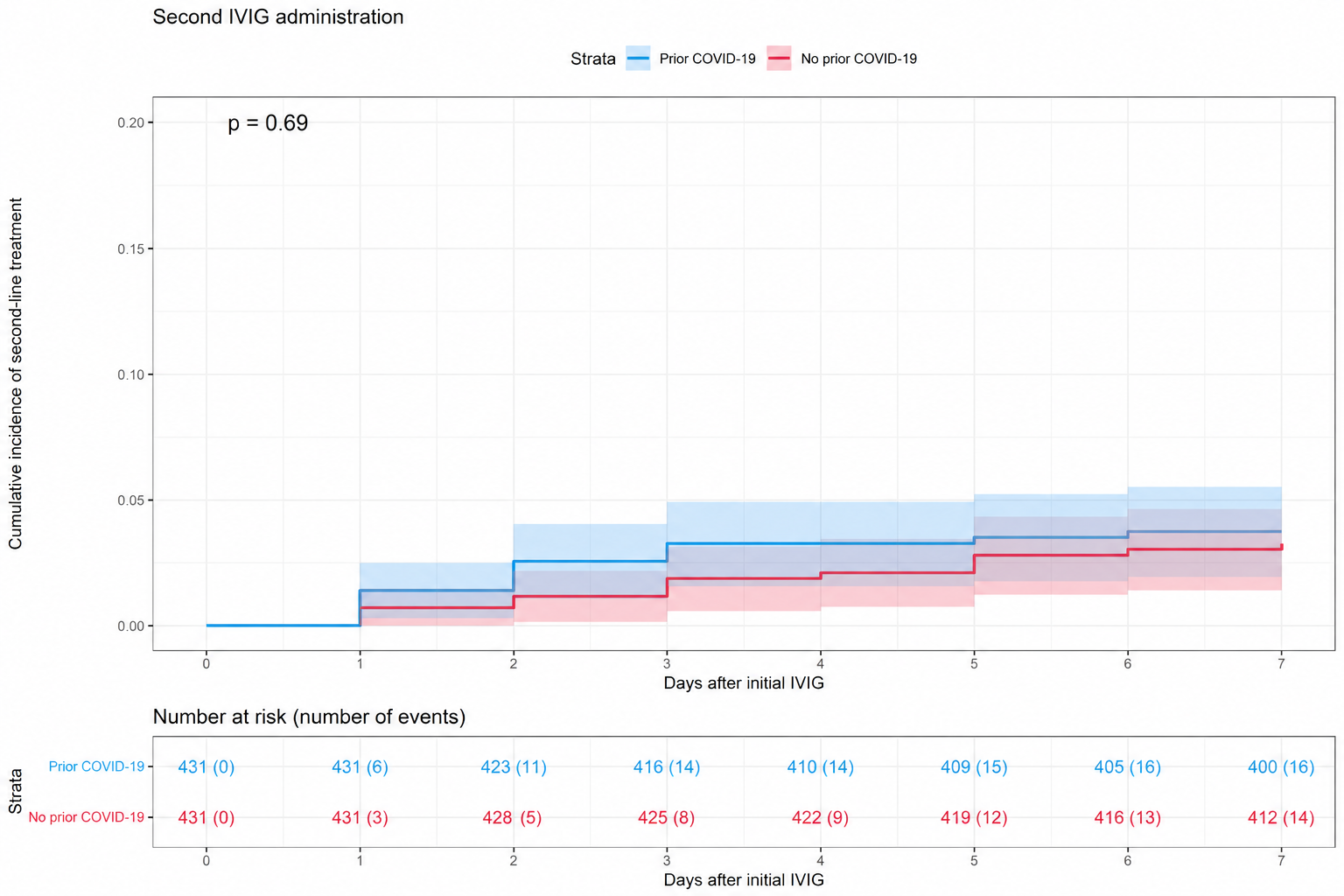


Cumulative incidence curves for second IVIG administration alone (one component of the composite second-line treatment endpoint) within 7 days after initial IVIG. The cumulative incidence did not differ significantly between children with prior COVID-19 (16 events among 431 patients) and matched controls (14 events among 431 patients) (log-rank P = 0.69). Shaded areas indicate 95% confidence intervals. Numbers at risk and cumulative numbers of events are shown below the plot.

**Supplementary Figure 2. Cumulative incidence of additional steroid treatment within 7 days according to prior COVID-19 status in the matched cohort.**


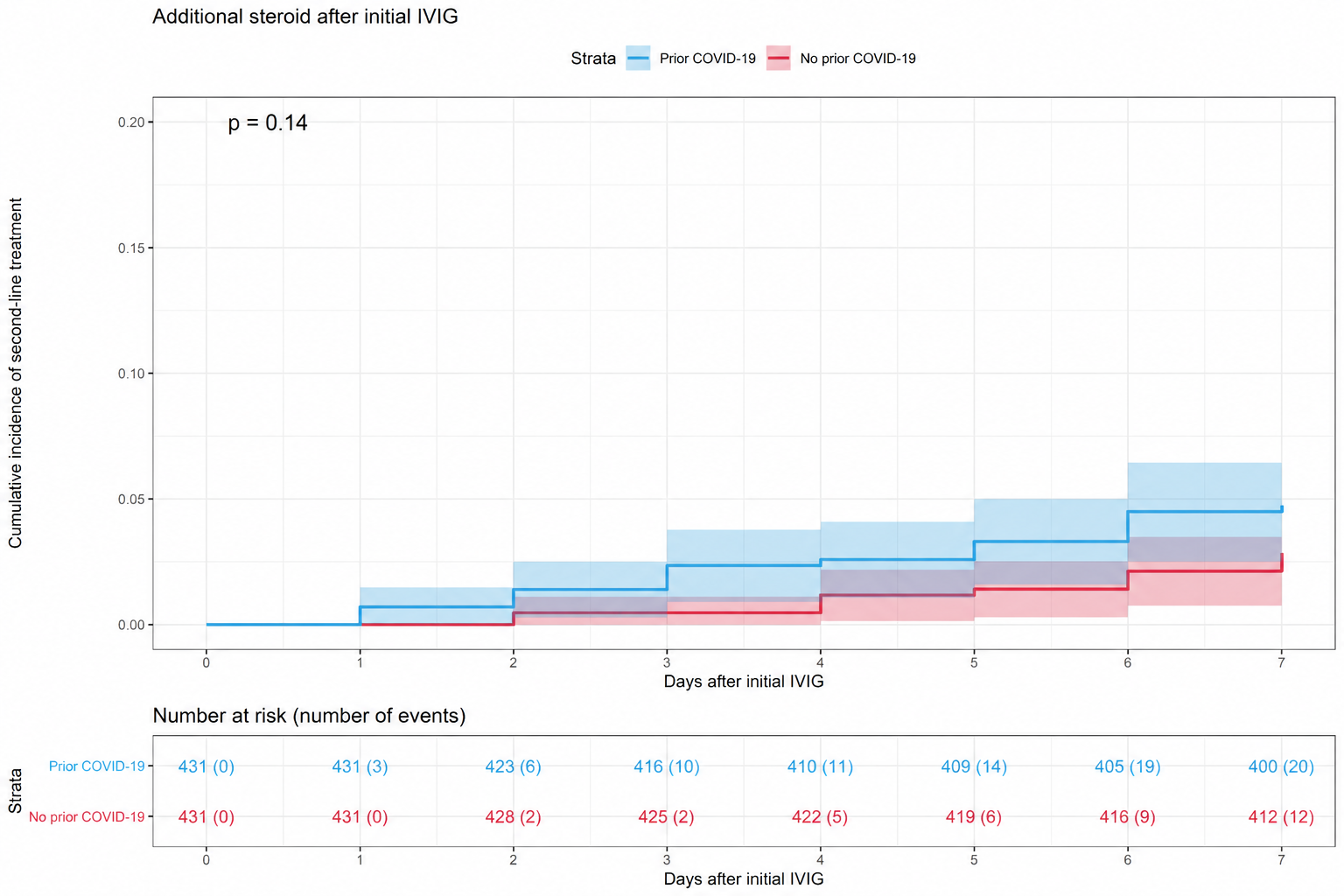


Cumulative incidence curves for additional steroid treatment alone (the other component of the composite second-line treatment endpoint), defined as systemic steroid administration on a date later than the initial IVIG. The cumulative incidence did not differ significantly between children with prior COVID-19 (20 events among 431 patients) and matched controls (12 events among 431 patients) (log-rank P = 0.14). Steroid use concurrent with initial IVIG was treated as a baseline characteristic and excluded from this outcome. Shaded areas indicate 95% confidence intervals.

**Supplementary Figure 3. Temporal trends in monthly COVID-19 and Kawasaki disease case counts during the study period.**


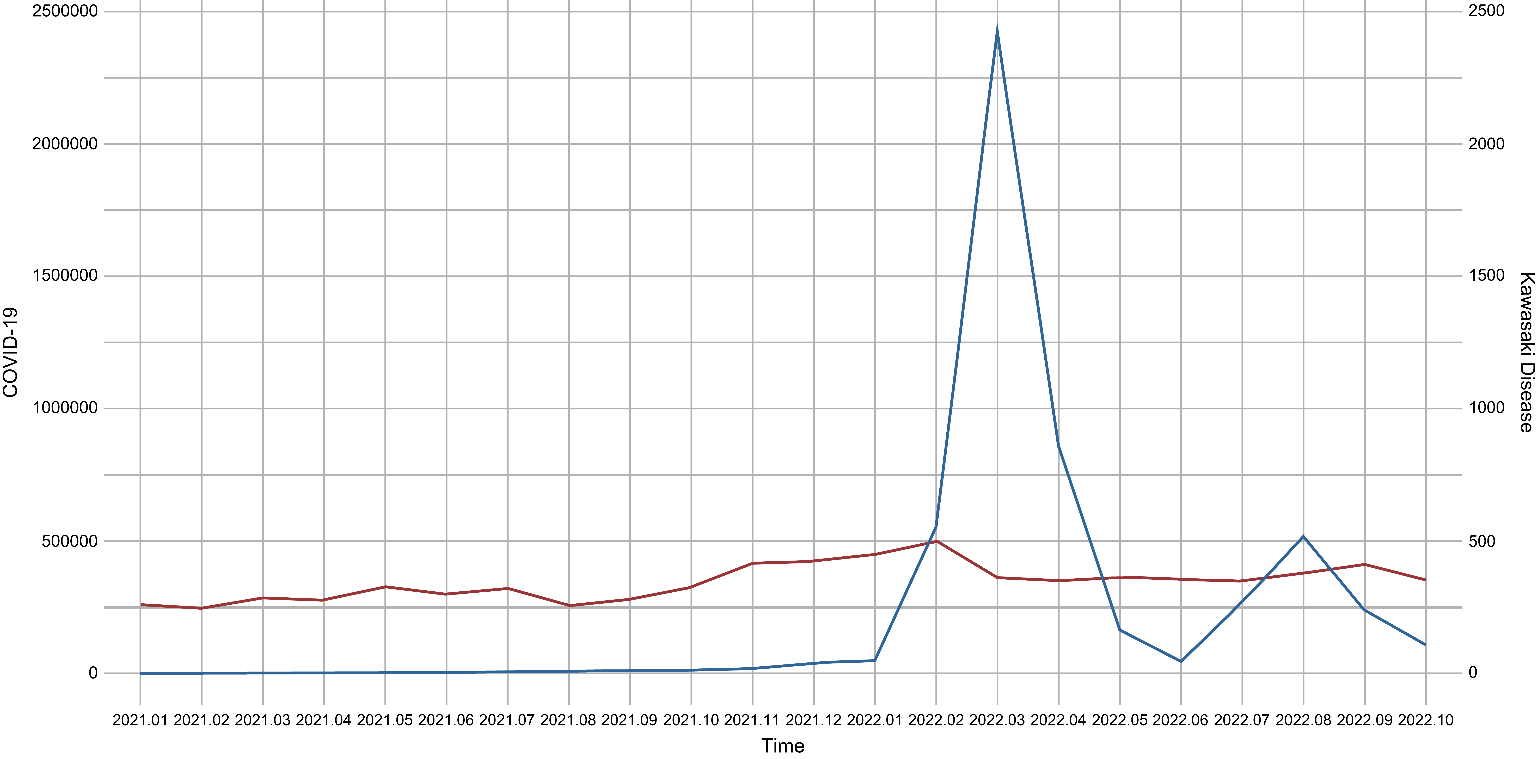


Monthly counts of newly reported COVID-19 cases (blue line, left y-axis) and Kawasaki disease cases (red line, right y-axis) in South Korea from January 2021 through October 2022. The Korean nationwide COVID-19 surveillance system showed marked variation across the study period, with a sharp peak in March 2022 corresponding to the Omicron-dominant wave. Kawasaki disease incidence remained relatively stable throughout the same period. This figure provides context for the sensitivity analysis restricted to the period from January 2022 onward (Supplementary Table 8), during which surveillance capture was most stable.
